# Evaluation of salivary lactate dehydrogenase level among tobacco users - A hospital based study

**DOI:** 10.6026/973206300213442

**Published:** 2025-10-31

**Authors:** Anvita Sinha, Nitesh Kumar Sharma, Sudhanshu Saxena, Anushree Prasad, Sonia Tiwari, Amit Raj, Sayantan Bhattacharya, Medha Krishnan, Amarta Lakra

**Affiliations:** 1 Department of Dentistry, M.G.M. Medical College and Hospital, Jamshedpur, Jharkhand, India; 2 Department of Conservative Dentistry and Endodontics, Awadh Dental College & Hospital, Jamshedpur, Jharkhand, India; 3 Department of Pediatric and Preventive Dentistry, Hazaribag College of Dental Sciences and Hospital, Hazaribag, Jharkhand, India; 4 Private Practitioner, Dant Aarogya Hospital, Biharsharif (Nalanda), Bihar, India; 5 Medical TCIL, Tata Steel Ltd, Jamshedpur, Jharkhand, India

**Keywords:** Biomarker, oral precancers, oral cancer, salivary lactate dehydrogenase, tobacco

## Abstract

Salivary lactate dehydrogenase (LDH) holds promise as a screening tool for early oral mucosal changes, including precancers and cancers, often linked to tobacco use. This study evaluated salivary LDH levels across 135 patients from the dental outpatient department at M. G. M. Medical College and Hospital, Jamshedpur, Jharkhand, India. Participants were categorized into three groups (n=45 each): non-tobacco users (controls), tobacco users without oral lesions and tobacco users with oral lesions. Unstimulated saliva samples were collected, centrifuged and analyzed for LDH levels. Results revealed significantly highest salivary LDH levels in tobacco users with lesions, followed by tobacco users without lesions and then the control group. These findings suggest that salivary LDH could be a valuable biomarker for detecting pathological changes in the oral mucosa of tobacco users.

## Background:

The great Indian Sikh Guru Gobind Singh had said, 'Wine is bad, Indian hemp (bhang) destroys one generation, but tobacco destroys all generations [1]. Even then globally, India is the third largest producer of tobacco and consumes around half of it. It has been used in a variety of ways in India [2]. Use of tobacco in smoke/smokeless forms has detrimental effects on oral health. It increases the risk of wide range of oral diseases such as oral mucosal lesions, periodontitis, oral potentially malignant disorders and oral cancer [3]. In the saliva, serum and tissues of an individual with various diseases, several biomarkers can be identified. Early identification of oral potential malignant disorders can stop the condition from progressing to oral cancer [4]. As an abundant enzyme and biomarker, lactate dehydrogenase (LDH) becomes detectable outside cells during cellular necrosis, cell death, or tissue degradation [5]. Studies suggest that the LDH in whole saliva is primarily non-glandular, with the oral epithelium being its principal source [6]. Since diverse pathological factors can compromise oral mucosal integrity, leading to lesions and oral squamous cell carcinoma, salivary LDH concentration may serve as a specific indicator of cellular damage [7]. Saliva as a clinical tool has a number of benefits over serum and tissues which includes non - invasiveness, ease in collection of samples, good co-operation with the patients, cost - effectiveness, easy storage, transportation, greater sensitivity and correlation with levels in blood [8]. Therefore, it is of interest to evaluate the levels of salivary Lactate Dehydrogenase enzyme in tobacco users with and without lesions and non- users. Also, to explore a simple, non- invasive, cost- effective, user- friendly, quick method of detecting tissue damage in tobacco users at the earliest.

## Methodology:

The present study was designed as a hospital based comparative study. Consecutive sampling was used to select the study population. Ethical approval for the research protocol was granted by the Institutional Ethics Committee of M. G. M. Medical College and Hospital, Jamshedpur, Jharkhand, India (Memo. No./IEC/07/22). The subjects included in the study were the patients aged between 18-70 years of both the genders, visiting the Dental Outpatient Department in M. G. M. Medical College & Hospital, Jamshedpur, Jharkhand, India. The individuals were explained about the purpose of the study and the prior written consent was obtained from those who voluntarily wanted to participate.

They were categorized into groups as follow:

[1] Group 1: Individuals without any tobacco habit (control group) (n = 45)

[2] Group 2: Individuals with tobacco habit without oral lesion (n= 45)

[3] Group 3: Individuals with tobacco habit with oral lesions (n = 45)

## Exclusion criteria:

[1] Individuals whose salivary characteristics could be altered by medication.

[2] Patients with history of systemic diseases known to increase serum LDH levels such as myocardial infarction, liver diseases, renal diseases, muscular dystrophy and cancer treatment.

## Collection of samples:

Participants were asked to refrain from eating, drinking, or smoking for an hour before sample collection. They sat upright in a dental chair with their head slightly tilted forward to facilitate saliva pooling. Morning samples (9-11 AM) were preferred to minimize diurnal variations. Two milliliters of unstimulated whole saliva were collected via the spit method into a sterile container under aseptic conditions, then immediately centrifuged at 5000 rpm for 5 minutes.

## Clinical examination:

Clinical examination was carried out by an experienced Oral Medicine specialist. Complete medical history and dental history of the patients were recorded. Both extraoral and intraoral examination was carried out wearing sterile hand gloves and mouth mask under artificial illumination. The patients' habit history was recorded in detail with reference to the presence or absence of tobacco, type, frequency and duration using specially designed preformed case sheet for the study. All the patients were educated about the ill effects of tobacco. Efforts were made to motivate tobacco users to discontinue their habit. In our study, all types of white and red lesions were recorded. No biopsy was performed on the cases for the study.

## Biochemical analysis:

An LDH - P kit (Erba Mannheim obtained from Tranasia Biomedical ltd., Solen, India) was used with an Ultraviolet spectrophotometer to carry out the assay. LDH levels in the samples collected were evaluated by the fully automatic analyzer. The assay was carried out within 24 hours. According to the manufacturer's procedural guidelines provided in the leaflet of the kit, the working reagent and the sample were mixed and the readings were recorded. It was in line with the recommendations by International Federation of Clinical Chemistry. Salivary LDH levels were quantified in IU/L.

## Statistical analysis:

Salivary LDH levels were summarized using mean, standard deviation, minimum, and maximum values for each group. As the Shapiro-Wilk test indicated non-normal data distribution, non-parametric Kruskal-Wallis and Mann-Whitney U tests were applied. A p-value < 0.05 signified statistical significance. All analyses were conducted using SPSS version 25 for Windows.

## Results:

Tobacco-related oral mucosal lesions identified were melanosis, smoker's palate, tobacco pouch keratosis, oral submucous fibrosis, and oral leukoplakia. All participants denied any history of alcohol use. Table 1 (see PDF) and Figure 1 (see PDF) show comparison of salivary LDH levels in different study groups. Mean ± SD of salivary LDH levels in Control, tobacco users without oral lesion and tobacco users with oral lesion groups were 407.44 ± 45.91 IU, 546.24 ± 107.59 IU and 1133.76 ± 369.43 IU respectively. Kruskal-Wallis test showed significant difference between the groups for salivary LDH levels (χ^2^ = 105.167, df = 2, P<0.001). After that Mann-Whitney U test was applied for pairwise comparison, which showed following observations: (1) Salivary LDH level in tobacco users with oral lesion group was significantly higher than control (MW = 0.000, P<0.001) and tobacco users without oral lesion groups (MW = 0.000, P<0.001). (2) Salivary LDH level in tobacco users without oral lesion group was significantly higher than control (MW = 0.000, P<0.001) group. The highest salivary LDH level was observed in tobacco users with oral lesion group followed by tobacco users without oral lesion group and control group.

## Discussion:

While invasive methods like tissue biopsies and blood tests remain the diagnostic gold standard, patient anxiety often reduces the uptake of crucial preventive screenings [9]. To address this, we chose saliva as our diagnostic medium. Saliva is readily available, non-invasive and offers a wealth of information, being less complex than blood and containing fewer inhibitory substances [10]. This makes it ideal for large-scale screenings and use in remote areas lacking specialized diagnostic facilities [6]. Salivary enzyme levels often change with oral mucosal pathologies [11]. LDH, a key enzyme in saliva, is one such indicator. It signals cellular injury and is a by-product of anaerobic glycolysis, existing in five isoforms: LDH-1, LDH-2, LDH-3, LDH-4 and LDH-5 [12]. For this study, we collected unstimulated saliva via the spit method, a commonly preferred technique [13, 14, 15- 16]. We included a group of tobacco users without existing oral potential malignant disorders (OPMD) or oral cancer to identify early biochemical changes and recognize individuals at high risk for whom preventive measures could be beneficial. LDH isoenzymes were not estimated due to the time-consuming and costly nature of that diagnostic method. Nagler *et al.* found that 75% of the oral fluid's salivary content came from oral epithelial cell desquamation, while only 25% originated from the salivary glands. So, the salivary LDH levels have the potential of biomarker of the diseased mucosa [7]. Therefore, the present study excluded participants with conditions known to non-specifically elevate both serum and salivary LDH, such as myocardial infarction, muscular dystrophy, and liver or renal diseases. A study comparing salivary and serum LDH in 30 healthy individuals and 30 smokers revealed significantly higher salivary LDH in smokers. This indicated saliva's superiority over serum for LDH estimation, confirming salivary LDH as an oral tissue damage marker [13]. Our study yielded similar results, though previous research exclusively focused on smokers, unlike our inclusion of smokeless tobacco users. Mantri *et al.* in a study on oral cancer, OSMF, tobacco chewers without lesions and healthy controls showed elevated salivary LDH activity in patients with tobacco pouch keratosis, OSMF and oral cancer [14]. This finding aligns with our study, which also reported significantly higher salivary LDH levels in tobacco users with oral lesions compared to both control participants and tobacco users without oral lesions. A study by Javariah *et al.* revealed a progressive rise in salivary LDH levels from control participants to tobacco users without oral lesions and further to those with oral lesions. Their findings suggested that salivary LDH could be a promising early biomarker for oral cancer [15]. Similarly, Mohan *et al.* compared LDH levels in serum and saliva among tobacco users and individuals with potentially malignant disorders. They found elevated salivary LDH in both groups compared to healthy controls. Additionally, Mohan *et al.* observed that salivary LDH levels were higher than serum LDH levels in tobacco users [16]. Elevated LDH activity primarily results from genomic changes during tissue destruction. Increased mitotic index and heightened lactic acid production by cells, stemming from glycoprotein breakdown, also contribute to these higher LDH levels [13]. Since elevated salivary LDH is observed with early oral mucosal damage, the current findings could help predict disease in tobacco users before clinical signs appear. Salivary LDH has been widely recognized as a diagnostic biomarker for oral potentially malignant disorders and oral cancers [4, 17, 18 and 19]. Despite this, establishing precise cut-off values remains a challenge. A key hurdle is the lack of a clearly defined normal range for salivary LDH in healthy individuals, as past studies have shown significant variations even within healthy populations. This inconsistency likely stems from a lack of standardized protocols for salivary LDH estimation, particularly regards saliva collection and analysis methods [18, 19]. Further research is strongly recommended to address these standardization issues.

## Conclusion:

The findings of the present study indicate that tobacco use is linked to increased salivary LDH levels, with the highest concentrations observed in individuals with oral lesions. Incorporating salivary diagnostics into tobacco awareness and cessation programs could be beneficial. Specifically, using salivary LDH levels might help motivate tobacco users to quit their habit.

## Source(s) of support:

Nil.

## Declaration on Publication Ethics:

The author's state that they adhere with COPE guidelines on publishing ethics as described elsewhere at https://publicationethics.org/. The authors also undertake that they are not associated with any other third party (governmental or non-governmental agencies) linking with any form of unethical issues connecting to this publication. The authors also declare that they are not withholding any information that is misleading to the publisher in regard to this article.

## Declaration on official E-mail:

The corresponding author declares that lifetime official e-mail from their institution is not available for all authors.
